# Early identification of peripheral neuropathy based on sudomotor dysfunction in Mexican patients with type 2 diabetes

**DOI:** 10.1186/s12883-019-1332-4

**Published:** 2019-05-31

**Authors:** Angelica Carbajal-Ramírez, Julián A. Hernández-Domínguez, Mario A. Molina-Ayala, María Magdalena Rojas-Uribe, Adolfo Chávez-Negrete

**Affiliations:** 1 Centro Médico Nacional Siglo XXI, Instituto Mexicano del Seguro Social (IMSS), Av. Cuauhtemoc 330, 06720 Mexico City, Mexico; 2 Mexican Social Security Institute, Mexico City, Mexico; 3 National Mexican Social Security Institute, Mexico City, Mexico

**Keywords:** MNSI, Electrochemical skin conductances, Small fiber neuropathies, Diabetic neuropathy

## Abstract

**Background:**

Type 2 Diabetes in Mexico has a high prevalence, 16–87% of patients may experience peripheral neuropathy. Early detection can prevent or halt its progression. The performance of Sudoscan in detecting neuropathy was compared to the Michigan Neuropathy Screening Instrument (MNSI). The aim was to identificate small fibers neuropathy.

**Methods:**

Patients type 2 diabetes received both MNSI and sudomotor function assessment through measurement of electrochemical skin conductance (ESC) in the hands and feet.

**Results:**

Two hundred twenty-one patients with neuropathy according to MNSI B had lower hands and feet ESC, regardless of diabetes duration. Among the 170 patients who had had diabetes for at least 5 years, 76 of them with normal MNSI B had abnormal hands or feet ESC; this was also the case in 28 out of 51 patients with diabetes than 5 or more years. In contrast, only 5 patients in the first group and 1 in the second group had abnormal MNSI B with normal ESC. Using MNSI B as a reference, abnormal hands or feet ESC (< 60 μS and 70 μS respectively) had a sensitivity of 97%, positive predictive value of 87% to detect neuropathy in patients with longer diabetes duration. The group with shorter diabetes duration, the sensitivity of abnormal hands or feet ESC to detect neuropathy was 91% while the positive predictive value was 88%.

**Conclusions:**

The Sudoscan device, which does not require any preparation, is noninvasive, easy and rapid to perform, can be useful in the early diagnosis peripheral neuropathy in type 2 diabetic.

## Background

Type 2 diabetes is now recognized as an immense and growing public health challenge worldwide, affecting about 382 million adults in 2014 and predicted to rise to 471 million by 2035 [1, 2]. Worryingly, in Mexico the prevalence is between 7.34% (2006 estimates) and 9.2%. According the ENSANUT survey in 2012 [3], 1.4% of diabetic patients received an amputation, underscoring the significant overall economic cost impact of the disease – $778,427,475 US dollars in 2011 [4].

The prevalence of diabetic peripheral neuropathy (DPN) varies according to different authors and instruments used for diagnosis; it is estimated to be between 16 and 87%. At time of diagnosis of type 2 diabetes, 7.5% of patients already have neuropathy [5–7] DPN has an annual incidence of 2% and is the most common neuropathy in industrialized countries [8]. Additionally DPN is a risk factor for complications such as diabetic foot – one out of every 5 patients presents with diabetic foot. In Mexico, 20% require foot amputation and 30% require a second amputation in the next 12 months [9].

Diabetic neuropathy encompasses a complex group of heterogeneous clinical manifestations, the most common being sensory-motor [10, 11]. DPN’s effects on autonomic function remain less investigated [12]. Studies suggest that the prevalence of autonomic cardiac neuropathy in diabetes ranges from 2.5% to as high as 90%, depending on the identifying criteria, and is often underdiagnosed [13, 14].

The clinical diagnosis of neuropathy using the Michigan Neuropathy Screening Instrument (MNSI) parts A (symptoms) and B (physical exam) is useful for its high sensitivity and specificity [15]. The consensus statement of the American Diabetes Association (ADA), the American Academy of Neurology (ANA) and Latin American Diabetes Association (ALAD) includes assessing sudomotor function’s role in the early diagnosis of autonomic neuropathy in diabetes [16, 17]. Sudoscan is a noninvasive, quick, easy, and reproducible method for the quantitative assessment of sudomotor function, through local measurements of electrochemical skin conductances [18–25].

We compared the results of Sudoscan and MNSI parts A and B for the identification of peripheral neuropathy in patients with type 2 diabetes, divided in two groups according to diabetes duration: less than 5 years and 5 years or more.

## Methods

The study was conducted at the Specialty Hospital of the National Medical Center Siglo XXI from March 2015 through February 2016, and was approved by the local research committee. Each patient signed a consent letter. Male and female adults aged 18 to 80 years, with or without symptoms of neuropathy, were enrolled. Two groups of patients were defined: those less than 5 years and those at least 5 years or more from diagnosis of type 2 diabetes according to ADA criteria. Patients who had undergone amputation of a limb were excluded. Medical history, weight, body mass index, and medications were recorded. We used the MNSI questionnaire to record patient responses regarding neuropathy and symptoms. For MNSI part B we performed a physical examination that included evaluation of skin changes, infection, muscle stretch reflexes, vibration sensation using a 128 Hz “*diapason*” on both feet, and pressure perception using a 10 g monofilament (Semmes-Weinstein monofilament Examination).

The evaluation of sudomotor function was measured with the Sudoscan medical device, consisting of a set of two electrodes for feet and hands connected to a computer. The duration of the test is 3 min on average, in which 4 combinations of 15 different low-voltage stimuli are applied. The patient does not require any preparation, and has only to place their palms and soles on the stainless-steel electrodes and remain for the test duration. The device measures the conductance generated in response to the electrical stimulus, which is expressed in micro Siemens for both the right and left side. It is a method based on stimulation of sweat glands by the low-level voltage, allowing evidence of sweat dysfunction not detectable under physiological conditions. No subject preparation is required for this test. Performance and accuracy of the method has been evaluated in numerous clinical studies [18–25].

Statistical analyses. Results for quantitative variables are shown as means ± SD. Quantitative variables were globally compared using ANOVA analysis. As a rule, a *p*-value < 0.05 was regarded as statistically significant. Performance of measurement of sudomotor dysfunction was assessed using Receiver Operating Curve (ROC) with calculation of the Area under the Curve (AUC). The data management and statistical analysis were done using SAS version 9.4 and R version 2.13.1 [26].

## Results

Of the 221 patients involved in the study, 51 had diabetes duration of 5 years o more. In the group patients were older, had lower BMI, and had more severe neuropathy according to MNSI B – they also had lower hands and feet ESC (Table 1).

**Table 1:** Characteristics of the study population

	Diabetes since Less than 5 years	Diabetes since 5 years or more	*p value* ^*¥*^
n	170	51	
Age (yrs)	58.6 ± 12.6	63.8 ± 11.8	*0.0095*
Gender, n (%)			*0.364*
Male	55 (32)	20 (39)	
Female	115 (68)	31 (61)	
BMI (kg/m^2^)	28.8 ± 6.2	27.2 ± 3.9	*0.0297*
Abnormal Michigan part B, n (%)	58 (34)	29 (57)	*0.0035*
Abnormal Feet ESC, n (%) ^a^	121 (71)	44 (86)	*0.1116*
Abnormal Hands ESC, n (%) ^a^	51 (30)	33 (65)	*< 0.0001*
Abnormal Hands or Feet ESC, n (%)^a^	129 (76)	43 (84)	*0.2036*
Feet ESC (μS)	58.6 ± 12.6	47.9 ± 20.2	*0.0009*
Hands ESC (μS)	64.5 ± 16.3	48.5 ± 21.5	*< 0.0001*

¥p value of Student test for means and chi^2^ test for percentages

^a^Abnormal feet ESC is defined as feet ESC < 70 μS and 60 μS for hands

**Fig. 1:**
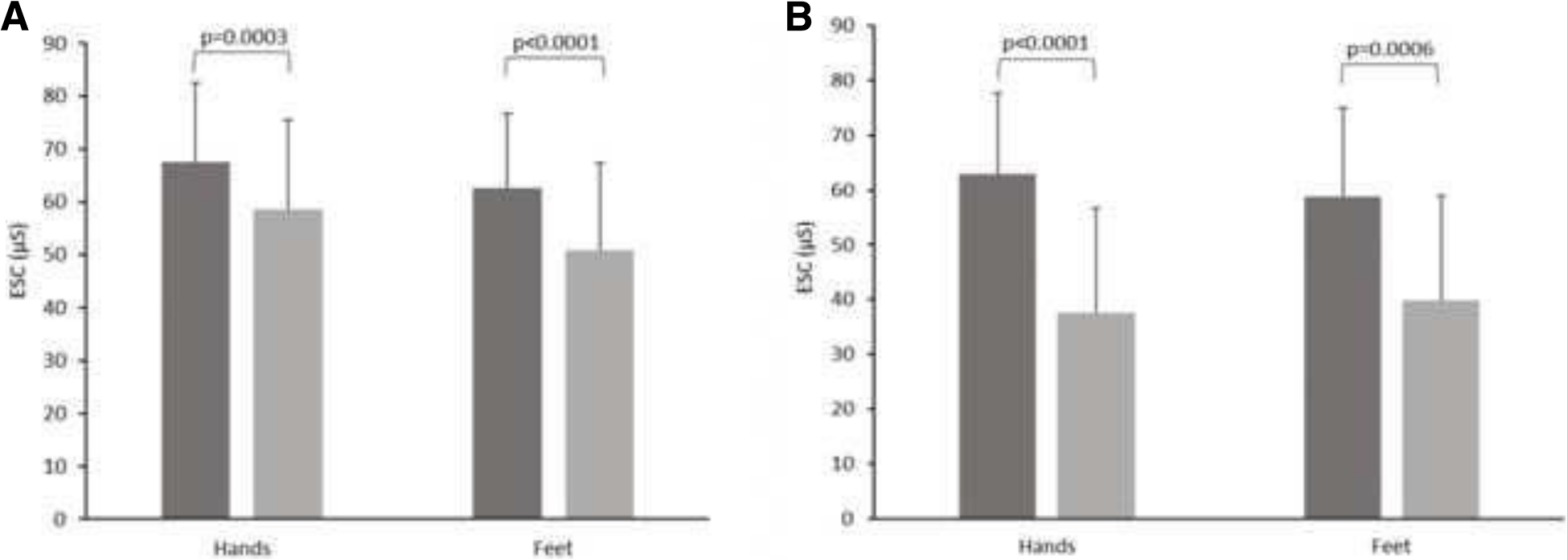
**a**. Distribution of hands and feet ESC according to MNSI (B) in type 2 diabetes patients since less than 5 years. In normal MNSI (B) group (left bar) mean ESC was 67.5 ± 15.0 μS for hands and 62.6 ± 14.1 μS for feet. In abnormal MNSI group (B) (right bar) it was 58.6 ± 17.1 μS and 50.9 ± 16.4 μS respectively. **b.** Distribution of hands and feet ESC according to MNSI (B) in type 2 diabetes patients since at least 5 years. In normal MNSI (B) group (left bar) mean ESC was 62.9 ± 14.8 μS for hands and 58.7 ± 16.3 μS for feet. In abnormal MNSI group (B) (right bar) it was 37.5 ± 19.3 μS and 39.8 ± 19.1 μS respectively

In both groups, patients with neuropathy according to MNSI B had lower hands and feet conductances (Fig. 1a and b). In both groups, more patients had abnormal ESC as compared to abnormal MNSI B (Tables 2 and 3).

**Table 2:** Means and median of SUDOSCAN measures according to MNSI(B) in diabetic patients < 5 years

	Normal Michigan B (*n* = 112)		Abnormal Michigan B (*n* = 58)		p value for means	p value for medians
	Mean ± STD	Median (Q1-Q3)	Mean ± STD	Median (Q1-Q3)		
Hands ESC (μS)	67.5 ± 15.0	72 (60–78)	58.6 ± 17.1	61 (49–71)	*0.0003*	*0.0008*
Feet ESC (μS)	62.6 ± 14.1	66 (55–73)	50.9 ± 16.4	53 (40–63)	*< 0.0001*	*< 0.0001*

*P* value of Wilcoxon test for means and χ^2^ for medians

**Table 3:** Means and median of SUDOSCAN measures according to MNSI(B) in diabetic patients ≧5 years

	Normal MNSI B (*n* = 22)		Abnormal MNSI B (*n* = 29)		p value for means	p value for medians
	Mean ± STD	Median (Q1-Q3)	Mean ± STD	Median (Q1-Q3)		
Hands ESC (μS)	62.9 ± 14.8	63 (50–76)	37.5 ± 19.3	33 (25–49)	*< 0.0001*	*0.0002*
Feet ESC (μS)	58.7 ± 16.3	59 (50–73)	39.8 ± 19.1	42 (24–53)	*0.0006*	*0.0035*

*P* value of Wilcoxon test for means and χ^2^ for medians

**Fig. 2:**
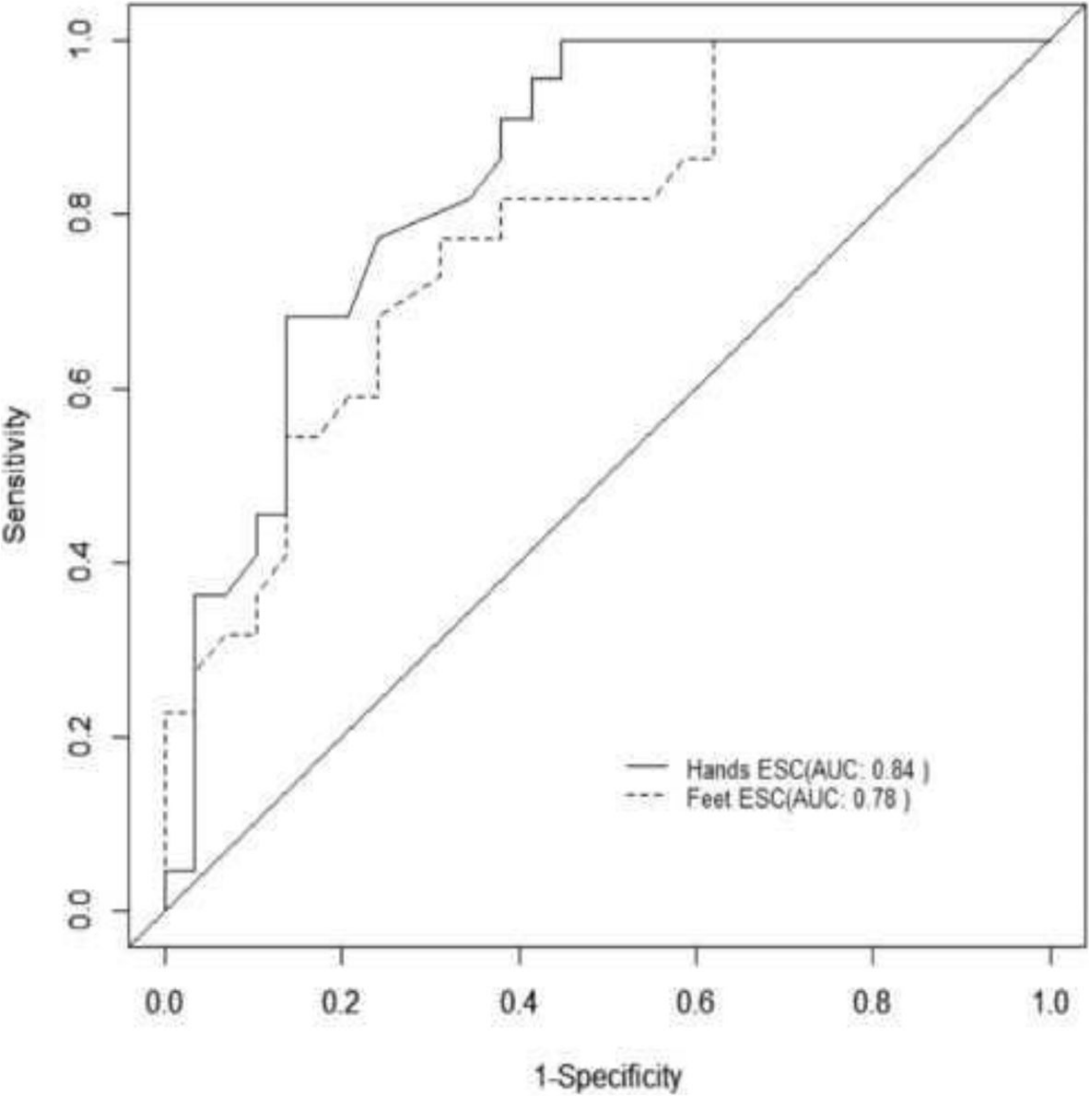
ROC curve for hands and feet ESC to detect neuropathy using MNSI (B) as gold standard in diabetic patients since less than 5 years

**Fig. 3:**
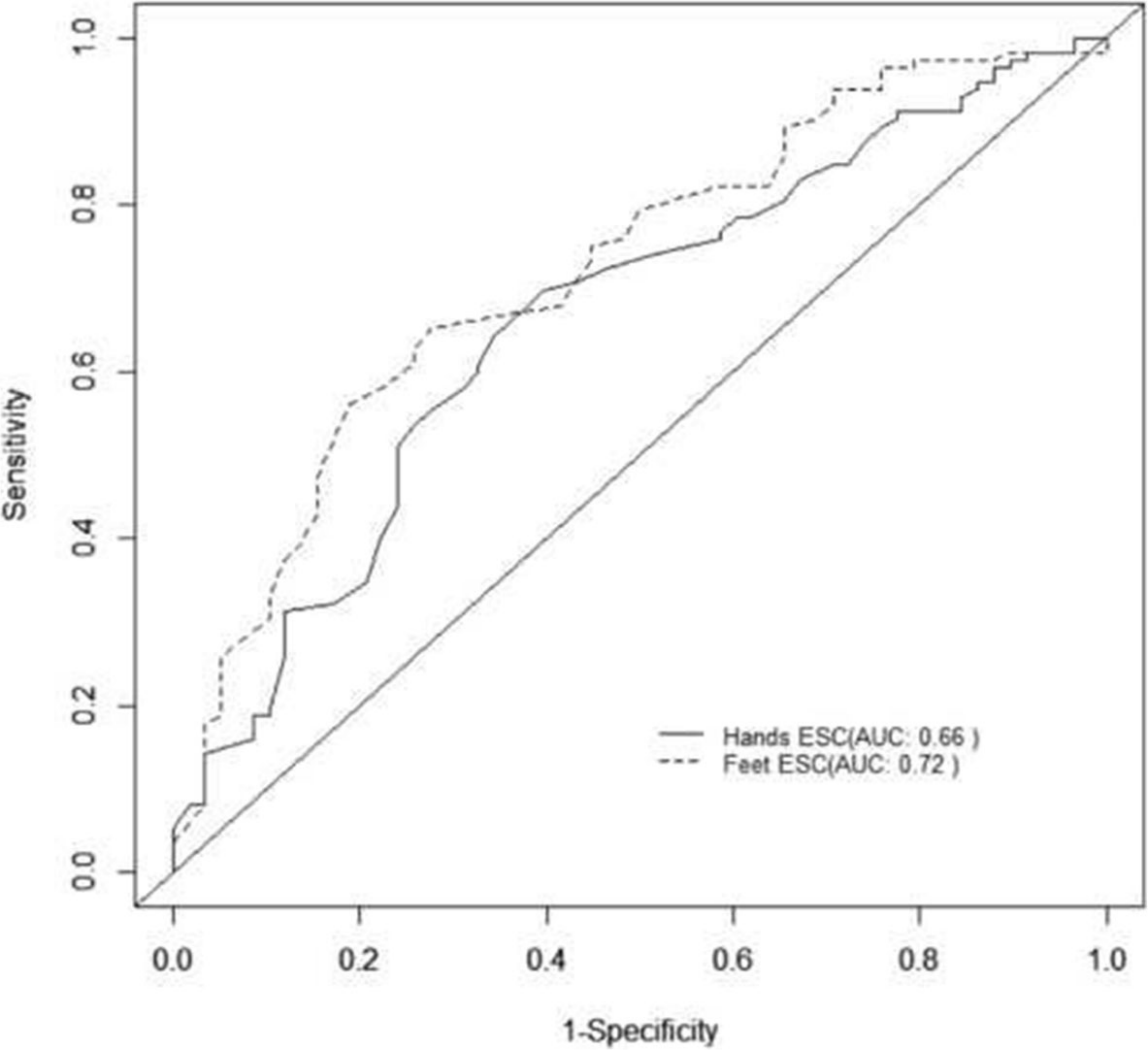
ROC curve for hands and feet ESC to detect neuropathy using MNSI (B) as gold standard in diabetic patients since at least 5 or more years

In particular, among the 170 patients with 5 or more years diabetes duration, 76 patients with normal MNSI B had abnormal hands or feet ESC, while this was true for 28 out of 51 in the other group. In contrast only 5 patients in the first group and 1 in the second group had abnormal MNSI B with normal ESC. Using MNSI B as a reference, hands or feet abnormal ESC (< 60 μS and 70 μS respectively) had a sensitivity of 97% and a positive predictive value (PPV) of 87% to detect neuropathy in patients with longer diabetes duration (Fig. 2). In patients with diabetes duration of less than 5 years, AUC of ROC curves for hands and feet ESC were 0.66 and 0.72 respectively (Fig. 3). The sensitivity of abnormal hands or feet ESC for detection of neuropathy was 91%, while the positive predictive value was 88% (Table 4).

**Table 4:** Chi^2^ tests between Sudoscan and MNSI

	MNSI (B)	
	Normal	Abnormal
*Diabetic patients since < 5 years*		
Hands and feet ESC both normal	36	5
At least one abnormal	76	53
	*p chi*^*2*^ *= 0.0007*	
*Diabetic patients since ≧ 5 years*		
Hands and feet ESC both normal	7	1
At least one abnormal	28	15
	*p chi*^*2*^ *= 0.0058*	

Any subject involved in the study reported neither adverse event nor discomfort during or after Sudoscan measurement.

## Discussion

This study demonstrated that i) hands and feet ESC decreased with diabetes duration; ii) patients with peripheral neuropathy according to MNSI B had lower hands and feet ESC, whatever their diabetes duration; iii) more patients had abnormal hands or feet ESC as compared to abnormal MNSI B; and iv) detection of neuropathy based on assessment of sudomotor function using MNSI B as a reference had a high performance, with a sensitivity of 97% and PPV of 87% in patients with diabetes duration of at least 5 years – sensitivity and PPV were 91 and 88% respectively in patients with diabetes duration of less than 5 years.

MNSI B is a sensitive screening instrument for routine evaluation of peripheral neuropathy in diabetic patients, as evidenced in many clinical studies, but it is time consuming in a busy clinical setting [15].

Early identification of subjects with DPN using novel, non-invasive methods may allow for intensified treatment of blood glucose and cardiovascular risk factors in order to prevent or halt the progression of DPN. This is critically important, as neuropathy is significantly associated with patient morbidity (foot ulceration, amputations, disabling pain, etc.) and mortality [5–8]. Therefore, the development of non-invasive, rapid and sensitive measures of neuropathy has a clinically sound rationale. Changes in peripheral autonomic nervous system function are an early manifestation of distal small fiber neuropathy [13, 14] Sweat glands are innervated by sudomotor, thin, unmyelinated sympathetic C-fibers and a number of skin biopsy studies have shown a reduction in the epidermal C-nerve fibers in patients with diabetes or pre-diabetes [27]. Sudomotor dysfunction is one of the earliest detectable abnormalities in distal small fiber neuropathies [12]. Therefore, assessment of sudomotor function may be an attractive tool to evaluate peripheral small fiber neuropathy in diabetes.

The performance of Sudoscan – which allows rapid, non-invasive, objective and quantitative assessment of sudomotor function – has been evaluated in several clinical studies performed in diabetic populations, using various tests as references. Sensitivities observed in the present study are higher or comparable to sensitivities observed in patients with type 1 or 2 diabetes by Casellini et al. (78% when compared to Neuropathy Impairment Score Lower Limb [NIS-LL] as reference); by Yajnik et al. (73% when using Vibration Perception Threshold as reference); and by Selvarajah et al. (88% when compared to combination of NIS-LL and nerve conduction studies). MNSI, like the tests used in previous studies, mainly assesses large fibers, while sudomotor dysfunction measurement assesses small fibers [18, 25]. Small fiber neuropathies can be observed earlier than large fiber neuropathy in type 2 diabetes, which can explain why in the present study more patients had abnormal ESC as compared to MNSI B [28]. This supports the relevance of this method of early detection. Meanwhile, comparable sensitivities were observed when Sudoscan was compared to methods which exclusively assess small fiber neuropathies, as observed by Lefaucheur et al., (76% in patients with confirmed peripheral neuropathy as assessed by reference methods: laser evoked potential [LEP] and quantitative sensory test [QST]), or to methods assessing large and small fiber neuropathies by Smith et al., (77% when compared to Utah Early Neuropathy Score [UENS]) [29, 21].

The present study has several limitations: i) patients were classified only by diabetes duration and only type 2 diabetes, ii) MNSI B was used as reference and comparator, iii) only sudomotor dysfunction assessment was used to assess small fiber neuropathy.

## Conclusions

Sudoscan should be considered to be in accordance with the recommendations of the ADA and ANA because it is rapid, easy to perform, robust, does not require any preparation, noninvasive, and reproducible. The evaluation of sudomotor function with this innovative tool is useful in the early identification of neuropathy, as it is able to detect damage to small fibers in patients with type 2 diabetes of any duration.

## Abbreviations

ADA: American Diabetes Association
ALAD: Latin American Diabetes Association
ANA: American Academy of Neurology
DPN: Diabetic peripheral neuropathy
ENSANUT: National Probabilistic Health Nutrition Survey
ESC: Electrochemical skin conductance
MNSI B: Part B of The Michigan Neuropathy Screening Instrument
MNSI: The Michigan Neuropathy Screening Instrument
NIS-LL: Neuropathy impairment score lower limb
UENS: Utah Early Neuropathy Score
